# Evaluation of the Clinical Systems for Polymyxin Susceptibility Testing of Clinical Gram-Negative Bacteria in China

**DOI:** 10.3389/fmicb.2020.610604

**Published:** 2021-02-22

**Authors:** Ying Zhu, Peiyao Jia, Menglan Zhou, Jingjia Zhang, Ge Zhang, Wei Kang, Simeng Duan, Tong Wang, Yingchun Xu, Qiwen Yang

**Affiliations:** ^1^Department of Clinical Laboratory, State Key Laboratory of Complex Severe and Rare Diseases, Peking Union Medical College Hospital, Chinese Academy of Medical Science and Peking Union Medical College, Beijing, China; ^2^Graduate School, Peking Union Medical College, Chinese Academy of Medical Sciences, Beijing, China

**Keywords:** antimicrobial susceptibility testing, polymyxin, essential agreement, category agreement, very major error, major error

## Abstract

**Objectives:**

The performance of mainstream commercial antimicrobial susceptibility testing systems on polymyxins has not been well evaluated in China. In this study, three antimicrobial susceptibility testing systems were evaluated for polymyxin B and colistin.

**Methods:**

The MICs of 257 Gram-negative strains collected from clinical cases and livestock were determined and analyzed. Using Broth Microdilution as the gold standard, the performance of VITEK 2^®^ COMPACT, Phoenix^TM^ M50, and Bio-kont AST System were evaluated. Essential agreement (EA), category agreement (CA), very major error (VME), and major error (ME) were calculated for comparison. The results of *mcr-1* positive strains were separately discussed.

**Results:**

The EA, CA, VME, and ME to polymyxin B for Bio-kont were 83.5, 95.6, 13.1, and 0.6%, respectively. The EAs, CAs, VMEs, and MEs to colistin were as follows: Bio-kont, 86.7%/96.5%/7.2%/1.7%; Vitek 2, 64.2%/86.8%/41.0%/0%, and Phoenix M50, 92.9%/92.9%/21.7%/0%. The performance of Bio-kont to polymyxin B and colistin for *Pseudomonas* spp. (EA, CA < 90%, VME > 1.5%, ME = 5.6%/10%) and *Enterobacter* spp. (EA, CA < 90%, VME > 1.5% and ME = 0%), Vitek to colistin for most genera, and Phoenix to colistin for *Enterobacter* spp. (EA, CA < 90%, VME > 1.5%, ME = 0%) were unsatisfactory compared with other genera. The performance of Bio-kont to polymyxins for *Escherichia* spp. and Phoenix to colistin for *Citrobacter* spp., *Escherichia* spp., and *Klebsiella* spp., which all met the CLSI standard, were satisfactory. When the susceptibility of *mcr-1* positive *E. coli* was tested, Bio-kont and Phoenix M50 presented excellent performance with no category errors, while Vitek 2 performed a high VME (25.5%).

**Conclusion:**

With relatively more accurate results for polymyxin B and colistin and lower VME, Bio-kont has an advantage in polymyxin antimicrobial susceptibility testing, especially for *Escherichia* spp., *Klebsiella* spp., *Citrobacter* spp. and *Acinetobacter* spp.

## Introduction

Polymyxin B and polymyxin E (colistin) are components of antibiotics produced by fermentation of *Paenibacillus polymyxa*. Despite the high incidence of nephrotoxicity and neurotoxicity associated with these agents, the family of polymyxins includes A, B, C, D, and E (Nation et al., 2014). Polymyxins have gained more attention in clinical practice due to the high susceptibility of multidrug-resistant (MDR) Gram-negative bacteria. Colistin and polymyxin B differ by just one amino acid in the peptide ring (Nation et al., 2014). Furthermore, polymyxin B is administered as an active form, while colistin in the form of an inactive prodrug, colistin methanesulfonate (CMS), which is also known as colistimethate (Tran et al., 2016).

Colistin or polymyxin B was first used in the 1950s for treating infections caused by Gram-negative MDR pathogens. These antibiotics fell out of favor and were replaced by agents with wider therapeutic indexes and less toxicity (Loho and Dharmayanti, 2015). The polymyxin class has recently re-emerged as a last-line agent in the treatment of multi-drug resistant pathogens non-susceptible to fluoroquinolones, aminoglycosides, and beta lactams (Bialvaei and Samadi Kafil, 2015). However, limited experience and lack of reliable consensus guidelines could potentially result in inappropriate use of these last-line antibiotics by clinicians.

As resistance to polymyxins has posed a great challenge in the treatment of infectious diseases, rapid and accurate detection of polymyxin-resistant strains is needed to prevent and control the outbreak of resistant strains. At present, the broth microdilution (BMD) method is the standard reference method for antimicrobial susceptibility testing (AST). However, the whole procedure is cumbersome and the testing requirements are strict, so it is rarely performed manually in clinical practice. At present, VITEK 2^®^ COMPACT (BioMérieux) and Phoenix^TM^ M50 (Becton Dickson Diagnostics) are the most commonly used AST systems in China. However, these systems have unsatisfactory performance when utilized to determine polymyxin susceptibilities. The purpose of this study is to evaluate the performance of three mainstream antimicrobial susceptibility systems in China on colistin and polymyxin B and to select the most accurate one.

## Materials and Methods

### Strains

A total of 257 non-repetitive Gram-negative strains were collected from clinical cases (202 *mcr-1* negative strains) and livestock (55 *mcr-1* positive strains) in China with different polymyxin MICs, including *Escherichia* spp. (*n* = 136), *Klebsiella* spp. (*n* = 27), *Citrobacter* spp. (*n* = 23), *Enterobacter* spp. (*n* = 25), *Acinetobacter* spp. (*n* = 23), and *Pseudomonas* spp. (*n* = 23). All strains were identified by the Vitek MS MALDI-TOF (BioMérieux) system.

### Antimicrobial Susceptibility Testing

BMD, the gold-standard reference method for AST, was performed strictly in accordance with the CLSI M7-A10 document (Clinical and Laboratory Standards Institute, 2015). The minimum inhibitory concentrations (MICs) of colistin or polymyxin B were measured only when the growth control was acceptable. *Escherichia coli* ATCC25922 and *Pseudomonas aeruginosa* ATCC27853 were used as quality controls. The performance of three commercial methods, including VITEK 2^®^ COMPACT (BioMérieux) with AST-N335 card (colistin), Phoenix^TM^ M50 (Becton Dickson Diagnostics) with NMIC-413 card (colistin), and Bio-kont AST System (Wenzhou Bio-kont) with polymyxin AST card (colistin and polymyxin B) were evaluated. The AST results of each test strain were considered accurate only when the MIC results of quality control strains were in the QC range.

### Data Analysis

MICs were analyzed according to the CLSI M100-E30 document (CLSI, 2020) and EUCAST clinical breakpoints-bacteria (v 10.0) (EUCAST, 2020). To date, the MIC of 2 mg/L has been the CLSI intermediate breakpoints of polymyxin B and colistin for Enterobacteriaceae, Pseudomonas, and Acinetobacter. Given that there is no breakpoint for the susceptible, results that were interpreted as “intermediate” were treated as “susceptible” in order to better calculate ME and VME, which is in accordance with the EUCAST standard. We interpreted the results in this manner: susceptible ≤2mg/L, resistance ≥4mg/L (Humphries et al., 2019).

Using BMD as the reference method, the following parameters are included in the assessment: Essential agreement (EA) was defined as a percentage of MICs measured by the system within ± 1 dilution of reference MICs. Category agreement (CA) represents the percentage of results with the same susceptibility categorization as BMD. Very major error (VME) stands for the percentage of false susceptible results compared to BMD. Major error (ME) means the percentage of false resistant results compared to BMD. According to CLSI recommendations, a new system can be acceptable when it meets the standards as follows: CA ≥ 90%, EA ≥ 90%, VME ≤ 1.5%, and ME ≤ 3% (CLSI, 2015).

## Results

### Antimicrobial Susceptibility Test

The MICs of quality control strains were all within the expected reference ranges specified by CLSI M100-S30 (CLSI, 2020). Susceptibilities to polymyxin B and colistin were tested in all 257 strains by BMD. Due to differences in AST cards, both drugs were not always tested by each system. Susceptibilities to polymyxin B were not reported by Vitek 2 and Phoenix M50. Due to loss in the tests, not all strains were reported by each system.

**FIGURE 1:**
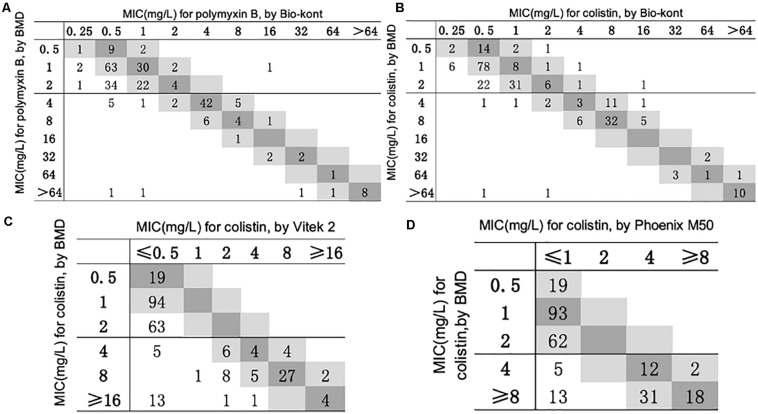
Scatterplot of three AST methods versus reference MIC obtained from BMD. The BMD MICs have been normalized in the scatterplots of Phoenix M50 and Vitek 2. When compared with Vitek 2, the maximum test limit is ≥ 16 mg/L, while compared with Phoenix M50, it is ≥ 8mg/L. **(A)** Scatterplot of Bio-kont versus reference MIC obtained from BMD for polymyxin B. **(B)** Scatterplot of Bio-kont versus reference MIC obtained from BMD for colistin. **(C)** Scatterplot of Vitek 2 versus reference MIC obtained from BMD for colistin. **(D)** Scatterplot of Phoenix M50 versus reference MIC obtained from BMD for colistin.

The general agreement between BMD and three AST systems for polymyxin B and colistin is shown in Figure 1. The performance of Bio-kont for polymyxin B and colistin both showed acceptable CAs (both 99.4%) and unacceptable EAs (77.8 and 82.2%, respectively) for susceptible strains identified by BMD. For resistant strains, the performances resulted in lower CAs (88.1 and 92.6%, respectively) and improved EAs (89.3 and 93.8%, respectively). The Vitek 2 system performed poorly with only 86.8% of results classified as CA and 62.6% classified as EA compared to the reference method. The performance of Phoenix M50 was better especially for susceptible strains with 100% results in both CA and EA. And for resistant strains, CA was 77.8% and EA was 70.4%.

The detailed statistics of three AST systems for 257 strains to polymyxin B and colistin are presented in Tables 1, 2.

**TABLE 1:** Comparison of performance characteristics to polymyxin B between Bio-kont AST System and BMD method for the seven genera in the 257 strains.

		Total	Polymyxin B					
Genera	Method		S	R	Performance [*n* (%)]			
					EA	CA	VME	ME
*Acinetobacter* spp.	BMD	23	22	1				
	Bio-kont	22	21	1	14 (53.7%)	22 (100%)	0 (0%)	0 (0%)
*Pseudomonas* spp.	BMD	23	18	5				
	Bio-kont	23	21	2	18 (78.33%)	18 (78.3%)	4 (80%)	1 (5.6%)
*Citrobacter* spp.	BMD	23	22	1				
	Bio-kont	23	23	0	17 (73.9%)	22 (95.7%)	1 (4.3%)	0 (0%)
*Enterobacter* spp.	BMD	25	10	15				
	Bio-kont	25	13	12	19 (76.0%)	22 (88.0%)	3 (12.0%)	0 (0%)
*Escherichia* spp.	BMD	136	78	58				
	Bio-kont	136	80	56	123 (90.4%)	134 (98.5%)	2 (1.5%)	0 (0%)
*Klebsiella* spp.	BMD	27	23	4				
	Bio-kont	26	22	4	22 (84.6%)	26 (100%)	0 (0%)	0 (0%)
Total	BMD	257	173	84				
	Bio-kont	255	180	75	213 (83.5%)	244 (95.6%)	10 (13.1%)	1 (0.6%)

*The interpretation rules: S (susceptible): MIC ≤ 2mg/L, R (resistance) MIC ≥ 4mg/L. EA (Essential agreement) = N_EA_^∗^100/N. CA (Category agreement) = N_CA_^∗^100/N. %VME (Very major error) = N_VME_^∗^100/total isolates resistance by BMD. %ME (Major error) = N_ME_^∗^100/total isolates susceptible by BMD.*

**TABLE 2:** Comparison of performance characteristics to colistin between each of the three AST systems and BMD method for the seven genera in the 257 strains.

Genera	Method	Total	Results		Performance [*n* (%)]			
			S	R	EA	CA	VME	ME
*Acinetobacter* spp.	BMD	23	21	2				
	Bio-kont	22	21	1	19 (86.4%)	21 (95.5%)	1 (50%)	0 (0%)
	Vitek	23	23	0	0 (0%)	21 (91.3%)	2 (100%)	0 (0%)
	Phoenix	22	22	0	20 (90.1%)	20 (90.9%)	2 (100%)	0 (0%)
*Pseudomonas* spp.	BMD	23	20	3				
	Bio-kont	23	20	3	19 (82.6%)	19 (82.6%)	2 (66.7%)	2 (10%)
	Vitek	23	23	0	4 (17.4%)	20 (87.0%)	3 (100%)	0 (0%)
	Phoenix	23	23	0	20 (87.0%)	20 (87.0%)	3 (100%)	0 (0%)
*Citrobacter* spp.	BMD	23	23	0				
	Bio-kont	23	22	1	15 (65.2%)	22 (95.7%)	0 (0%)	1 (4.3%)
	Vitek	23	23	0	14 (60.9%)	23 (100%)	0 (0%)	0 (0%)
	Phoenix	23	23	0	23 (100%)	23 (100%)	0 (0%)	0 (0%)
*Enterobacter* spp.	BMD	25	10	15				
	Bio-kont	25	13	12	19 (76.0%)	22 (88.0%)	3 (20.0%)	0 (0%)
	Vitek	25	24	1	8 (32%)	11 (44.0%)	14 (93.3%)	0 (0%)
	Phoenix	25	23	2	12 (48%)	12 (48.0%)	13 (86.7%)	0 (0%)
*Escherichia* spp.	BMD	136	79	57				
	Bio-kont	136	79	57	129 (94.9%)	136 (100%)	0 (0%)	0 (0%)
	Vitek	136	94	42	122 (89.7%)	121 (89.0%)	15 (26.3%)	0 (0%)
	Phoenix	136	79	57	136 (100%)	136 (100%)	0 (0%)	0 (0%)
*Klebsiella* spp.	BMD	27	23	4				
	Bio-kont	26	22	4	20 (76.9%)	26 (100%)	0 (0%)	0 (0%)
	Vitek	27	23	4	17 (63.0%)	27 (100%)	0 (0%)	0 (0%)
	Phoenix	26	22	4	26 (100%)	26 (100%)	0 (0%)	0 (0%)
Total	BMD	257	176	81				
	Bio-kont	255	177	78	221 (86.7%)	246 (96.5%)	6 (7.2%)	3 (1.7%)
	Vitek	257	210	47	165 (64.2%)	223 (86.8%)	34 (41.0%)	0 (0%)
	Phoenix	255	192	63	237 (92.9%)	237 (92.9%)	18 (21.7%)	0 (0%)

*The interpretation rules: S (susceptible): MIC ≤ 2mg/L, R (resistance) MIC ≥ 4mg/L. EA (Essential agreement) = N_EA_^∗^100/N. CA (Category agreement) = N_CA_^∗^100/N. %VME (Very major error) = N_VME_^∗^100/total isolates resistance by BMD. %ME (Major error) = N_ME_^∗^100/total isolates susceptible by BMD.*

As for polymyxin B, the Bio-kont AST System showed a CA ≥ 90% and a ME = 0% for most genera except *Pseudomonas* spp. and *Enterobacter* spp. However, immense diversity in EA was observed from 53.7 to 90.4% and high VMEs were showed in *Pseudomonas* spp. (80%), *Citrobacter* spp. (4.3%), and *Enterobacter* spp. (12%). The only species meeting the requirement of an acceptable system (CA = 90.4%, EA = 98.5%, VME = 1.5%, and ME = 0%) was *Escherichia* spp. against polymyxin B. EA for strains of *Acinetobacter* spp. for polymyxin B was merely 53.7%, though with high CA of 100%. The MICs of these strains tested by Bio-kont AST System are generally lower than those of BMD. The total EA rate of Bio-kont for polymyxin B was 83.5%, while the CA rate was 95.6%. Additionally, several VMEs (13.1%) and few ME (0.6%) were observed. CA and ME of Bio-kont met the required standards, and EA and VME did not meet the standards.

As for colistin, compared with BMD, the total EA was 86.7% for Bio-kont, 64.2% for Vitek 2, and 92.9% for Phoenix M50. Among the three AST systems, Vitek 2 showed the worst performance with the lowest EAs for each genus tested in the study, especially in *Acinetobacter* spp., *Pseudomonas* spp., and *Enterobacter* spp. In general, Phoenix M50 presented better EAs, followed by Bio-kont. The total CA was 96.5% for Bio-kont, 86.8% for Vitek 2 and 92.9% for Phoenix M50. Of particular attention, the systems yielded numerous VMEs: 7.2% for Bio-kont, 41.0% for Phoenix M50, and 21.7% for Vitek 2, mainly in *Acinetobacter* spp., *Pseudomonas* spp. and *Enterobacter* spp. along with a high VME (26.3%) from Vitek 2 in *Escherichia* spp. Very limited ME rates of 1.7% from Bio-kont and 0% from both Vitek 2 and Phoenix M50 were observed. The respective MEs were all 0% except *Pseudomonas* spp. from Bio-kont (10%) and *Citrobacter* spp. from Bio-kont (4.3%). On the whole, only the EA of Phoenix M50, CAs of Bio-kont and Phoenix M50, and MEs of three systems met the required standards.

To be more specific, although CAs were all >90%, the particularly high VMEs of *Acinetobacter* spp. from three systems were worth exploring. Additionally, the performance characteristics of *Pseudomonas* spp. to colistin were barely satisfactory with low EAs (Bio-kont 82.6%, Vitek 2 17.4%, Phoenix M50 87.0%), low CAs (Bio-kont both 82.6%, Vitek 2 and Phoenix M50 both 87.0%), and high VMEs (Bio-kont 66.7%, Vitek 2 and Phoenix M50 both 100%) and MEs (Bio-kont 10%, Vitek 2 0%, Phoenix M50 0%). The similar unsatisfactory performance of *Enterobacter* spp. to colistin was presented (low EAs: Bio-kont 76.0%, Vitek 2 32%, Phoenix M50 48%, low CAs: Bio-kont 88.0%, Vitek 2 44.0%, Phoenix M50 48.0%; high VMEs: Bio-kont 20.0%, Vitek 2 93.3%, Phoenix M50 86.7%).

The EAs for *Escherichia* spp. to colistin were highest (Bio-kont 94.9%, Vitek 2 89.7%, Phoenix M50 100%). No VMEs or MEs for *Escherichia* spp. to colistin from Bio-kont and Phoenix M50 were observed to have fully consistent agreement with BMD, which indicated the agreement of Bio-kont and Phoenix M50 with the rules (CA ≥ 90%, EA ≥ 90%, VME ≤ 1.5%, ME ≤ 3%).

Except for EAs, the performance characteristics of Vitek 2 and Phoenix M50 were similar in terms of colistin in three genera (for *Acinetobacter* spp.: CA ≥ 90%, VME = 100%, and ME = 0%; for *Citrobacter* spp. and *Klebsiella* spp.: CA = 100%, VME = 0%, ME = 0%).

In all, Bio-kont presented the best performance in the tested genus. However, the performance of *Pseudomonas* spp. and *Enterobacter* spp. requires further research and a larger sample size.

### Detection of *mcr*-1 Gene and Performance Evaluation on *mcr-1*-Positive/Negative Strains

Among 257 Gram-negative bacteria strains, 55 strains were positive for *mcr*-1 gene and all of them were *E. coli*. As *mcr-1* gene strongly indicates the resistance of colistin and polymyxin B, the susceptibilities of 55 *mcr-1-*positive strains are verified according to BMD. The MIC distribution of 55 *E. coli* strains is as follows. For polymyxin B, the MICs of 45 *mcr-1*-positive strains were 4 mg/L and those of 10 strains were 8 mg/L. For colistin, the MICs of 12 strains were 4 mg/L and those of 43 strains were 8 mg/L. All *mcr-1*-positive strains were low-level resistant to colistin (MIC: 4–8 mg/L) and polymyxin B (MIC: 4–8 mg/L). These strains can be classified as colistin-resistant and polymyxin B-resistant strains according to either CLSI standard document (CLSI, 2020) or EUCAST standard (EUCAST, 2020), which corresponds to the genomic explanation.

Utilizing BMD as standard, the performance of three AST systems for 55 *mcr-1* positive *E. coli* strains and other *mcr-1-*negative strains to polymyxin B and colistin are presented in Table 3.

**TABLE 3:** Comparison of performance characteristics to colistin and polymyxin B between each of the three AST systems and BMD method for strains of different *mcr-1* genetic conditions.

	*mcr-1* gene	Method	Total	S	R	Performance [*n* (%)]			
						EA	CA	VME	ME
Polymyxin B	+	BMD	55	0	55				
		Bio-kont	55	0	55	55 (100%)	55 (100%)	0 (0%)	0 (0%)
Colistin	+	BMD	55	0	55				
		Bio-kont	55	0	55	55 (100%)	55 (100%)	0 (0%)	0 (0%)
		Vitek	55	14	41	47 (85.5%)	41 (74.5%)	14 (25.5%)	0 (0%)
		Phoenix	55	0	55	55 (100%)	55 (100%)	0 (0%)	0 (0%)
Polymyxin B	-	BMD	202	173	29				
		Bio-kont	200	180	20	158 (79.0%)	189 (94.5%)	10 (50%)	1 (0.6%)
Colistin	-	BMD	202	174	28				
		Bio-kont	200	174	23	166 (83%)	191 (95.5%)	6 (21.4%)	3 (1.7%)
		Vitek	202	196	6	118 (58.4%)	182 (90.1%)	20 (71.4%)	0 (0%)
		Phoenix	200	192	8	182 (91%)	182 (91.0%)	18 (64.3%)	0 (0%)

*The interpretation rules: S (susceptible): MIC ≤ 2mg/L, R (resistance) MIC ≥ 4mg/L. EA (Essential agreement) = N_EA_^∗^100/N. CA (Category agreement) = N_CA_^∗^100/N. %VME (Very major error) = N_VME_^∗^100/total isolates resistance by BMD. %ME (Major error) = N_ME_^∗^100/total isolates susceptible by BMD.*

Compared with BMD, Bio-kont system and Phoenix M50 system presented excellent performance with no category errors when the susceptibility of *mcr-1*-positive *E. coli* was tested. It was worth noting that high VME rates (14/55, 25.5%) were observed from Vitek 2 system in *mcr-1*-positive *E. coli* strains. As for *mcr-1*-negative strains, each system showed acceptable rates of CA with several errors. The performance of Bio-kont was better than the other two systems with fewer errors. Noticeably high error rates were presented in both Vitek 2 system and Phoenix M50 system in *mcr-1*-negative strains. In conclusion, influence from *mcr-1* gene to Bio-kont and Phoenix M50 is minor, while that to Vitek 2 maybe need more data for analysis.

## Discussion

In this study, we evaluated the performance of three systems: Vitek 2, Phoenix M50, and Bio-kont. Vitek 2 and Phoenix M50 are two of the most commonly used AST systems in China while Bio-kont is a newly developed Chinese system that has obtained a marketing license. A joint EUCAST and CLSI polymyxin breakpoint working group recommended standard BMD as the reference method for the MIC testing of colistin (Carretto et al., 2018; Tsuji et al., 2019). However, diversity in instrument manufacturers, software stability, and even AST panels with different concentration gradients could make a difference in MIC results. With the preclinical PK/PD, clinical PK/TD, and MIC distribution data reviewed, the category of susceptibility was deemed to be inappropriate by CLSI, and an intermediate-only category was established as this category identifies isolates “that approach usually attainable blood and tissue levels and/or for which response rates may be lower than for susceptible isolates” (CLSI, 2020; Satlin et al., 2020). The absence of a susceptible category of the CLSI standard (CLSI, 2020) promoted us to cast light on the analysis referred to EUCAST standard (EUCAST, 2020).

Based on our study, none of the systems in this study met the standards for colistin and polymyxin B AST compared to BMD. Generally, Bio-kont was the most satisfactory system with the highest CA and least errors; it was followed by Phoenix M50, which had higher EA (92.9%), acceptable CA (92.9%), but more errors (21.7%). Vitek 2 showed the worst performance with low EA (64.2%), unacceptable CA (86.8%), and unexpectedly high VME (41.0%). The performances of the Vitek 2 and Phoenix M50 systems for colistin susceptibility test have been estimated before by Vourli et al. with a similar CA as this study (89.7% vs. 86.8% for Vitek 2 and 88.9% vs. 92.9% for Phoenix M50) (Vourli et al., 2017). In a study conducted by Ka Lip Chew et al., a VME rate of 36% for colistin testing by Vitek 2 was demonstrated (Chew et al., 2017). However, research into the Bio-kont system is limited as it is mainly applied in China. The evolution results of this system in this study has proved that this system—with satisfactory CA and an acceptable error rate—has better promotion value.

The problem of considerable errors in the detection of *Pseudomonas* spp. and *Enterobacter* spp. existed in all systems. In terms of *Pseudomonas* spp., the MICs of these error-prone strains were 4 mg/L which were interpreted as ATU (EUCAST) or R—the dividing value (CLSI). It is worth mentioning that the error-prone *Enterobacter* spp. strains tested by Vitek 2 and Phoenix M50 were all highly resistant to colistin (MIC>64 mg/L). False susceptibility in polymyxin B for *Enterobacteriaceae* from Vitek 2 and Phoenix M50 has been reported before, which was suspected to be the result of a smaller inoculum size effect (Doern et al., 2011; Lat et al., 2011; Bobenchik et al., 2015; Zhou et al., 2018). Currently, the CLSI still felt it important to acknowledge that available data suggested limited clinical effectiveness of the polymyxins for the *Enterobacterales*, *P. aeruginosa*, and *Acinetobacter* spp. (Satlin et al., 2020). The results of this study require validation through further research.

The notable errors in *Escherichia* spp. from Vitek 2 were mainly from *mcr-1*-positive strains. In 2017, colistin was formally forbidden from animal feeds in China (China, 2017). Whether poor reliability of colistin susceptibility testing by Vitek 2 for *mcr-1*-positive *E. coli* is an objective existence or an occasional occurrence remains unknown.

Meanwhile, it could be observed that the MICs of colistin in several susceptible isolates tested by the Vitek 2 system were one- to two-fold dilutions lower than those of BMD, indicating that strains classified as S and I by Vitek 2 system should be verified by BMD (Vourli et al., 2017). More studies are needed to further interpret the poor performance of systems and to support the interpretation of AST results.

## Data Availability Statement

The original contributions presented in the study are included in the article/supplementary material, further inquiries can be directed to the corresponding author/s.

## Ethics Statement

The Human Research Ethics Committee of Peking Union Medical College Hospital approved this study and waived the need for consent (Ethics Approval Number: S-K771).

## Author Contributions

YZ wrote the manuscript. PJ, MZ, QY, and YX revised the manuscript. JZ, GZ, WK, SD, and TW performed the experiments. All authors approved the final version of the manuscript.

## Conflict of Interest

The authors declare that the research was conducted in the absence of any commercial or financial relationships that could be construed as a potential conflict of interest.

## References

[B1] Bialvaei A. Z. Samadi Kafil H. (2015). Colistin, mechanisms and prevalence of resistance. *Curr. Med. Res. Opin.* 31 707–721. 10.1185/03007995.2015.1018989 25697677

[B2] Bobenchik A. M. Deak E. Hindler J. A. Charlton C. L. Humphries R. M. (2015). Performance of Vitek 2 for antimicrobial susceptibility testing of *Enterobacteriaceae* with Vitek 2 (2009 FDA) and 2014 CLSI breakpoints. *J. Clin. Microbiol.* 53 816–823. 10.1128/JCM.02697-14 25540403 PMC4390649

[B3] Carretto E. Brovarone F. Russello G. Nardini P. El-Bouseary M. M. Aboklaish A. F. (2018). Clinical validation of SensiTest colistin, a broth microdilution-based method to evaluate colistin MICs. *J. Clin. Microbiol.* 56 e1523–17. 10.1128/JCM.01523-17 29343542 PMC5869822

[B4] Chew K. L. La M. V. Lin R. T. P. Teo J. W. P. (2017). Colistin and polymyxin B susceptibility testing for carbapenem-resistant and mcr-positive *Enterobacteriaceae*: comparison of sensititre, microscan, Vitek 2, and Etest with broth microdilution. *J. Clin. Microbiol.* 55 2609–2616. 10.1128/JCM.00268-17 28592552 PMC5648698

[B5] China (2017). *Announcement of the Ministry of Agriculture of the People’s Republic of China No. 2428.* Available online at: http://www.moa.gov.cn/nybgb/2016/dibaqi/201712/t20171219_6102822.htm (accessed December 01, 2017)

[B6] Clinical and Laboratory Standards Institute (2015). *Methods for Dilution Antimicrobial Susceptibility Test for Bacteria That Grow Aerobically Document in M07-A9*, 10th Edn. Wayne, PA: Clinical and Laboratory Standards Institute.

[B7] CLSI (2015). *Verification of Commercial Microbial Identification and Antimicrobial Susceptibility Testing Systems–First Edition: M52-Ed1.* Wayne, PA: Clinical and Laboratory Standards Institute.

[B8] CLSI (2020). *Performance Standards for Antimicrobial Susceptibility Testing, Twenty-Fifth Informational Supplement in M100-S30.* Wayne, PA: Clinical and Laboratory Standards Institute.

[B9] Doern C. D. Dunne W. M. Jr. Burnham C. A. (2011). Detection of *Klebsiella pneumoniae* carbapenemase (KPC) production in non-*Klebsiella pneumoniae Enterobacteriaceae* isolates by use of the Phoenix, Vitek 2, and disk diffusion methods. *J. Clin. Microbiol.* 49 1143–1147. 10.1128/JCM.02163-10 21209164 PMC3067756

[B10] EUCAST (2020). *EUCAST Clinical Breakpoints – Bacteria (v 10.0).* Växjö: European Committee on Antimicrobial Susceptibility Testing.

[B11] Humphries R. M. Green D. A. Schuetz A. N. Bergman Y. Lewis S. Yee R. (2019). Multicenter evaluation of colistin broth disk elution and colistin agar test: a report from the clinical and laboratory standards institute. *J. Clin. Microbiol.* 57 e1269–19. 10.1128/JCM.01269-19 31511331 PMC6813006

[B12] Lat A. Clock S. A. Wu F. Whittier S. Della-Latta P. Fauntleroy K. (2011). Comparison of polymyxin B, tigecycline, cefepime, and meropenem MICs for KPC-producing *Klebsiella pneumoniae* by broth microdilution, Vitek 2, and Etest. *J. Clin. Microbiol.* 49 1795–1798. 10.1128/JCM.02534-10 21367993 PMC3122677

[B13] Loho T. Dharmayanti A. (2015). Colistin: an antibiotic and its role in multiresistant Gram-negative infections. *Acta Med. Indones.* 47 157–168. 26260559

[B14] Nation R. L. Velkov T. Li J. (2014). Colistin and polymyxin B: peas in a pod, or chalk and cheese? *Clin. Infect. Dis.* 59 88–94. 10.1093/cid/ciu213 24700659 PMC4305129

[B15] Satlin M. J. Lewis J. S. Weinstein M. P. Patel J. Humphries R. M. Kahlmeter G. (2020). Clinical and Laboratory Standards Institute (CLSI) and European Committee on Antimicrobial Susceptibility Testing (EUCAST) position statements on polymyxin B and colistin clinical breakpoints. *Clin. Infect. Dis.* 71 e523–e529. 10.1093/cid/ciaa121 32052041

[B16] Tran T. B. Velkov T. Nation R. L. Forrest A. Tsuji B. T. Bergen P. J. (2016). Pharmacokinetics/pharmacodynamics of colistin and polymyxin B: are we there yet? *Int. J. Antimicrob. Agents* 48 592–597. 10.1016/j.ijantimicag.2016.09.010 27793510 PMC5154767

[B17] Tsuji B. T. Pogue J. M. Zavascki A. P. Paul M. Daikos G. L. Forrest A. (2019). International consensus guidelines for the optimal use of the polymyxins: endorsed by the American College of Clinical Pharmacy (ACCP), European Society of Clinical Microbiology and Infectious Diseases (ESCMID), Infectious Diseases Society of America (IDSA), International Society for Anti−infective Pharmacology (ISAP), Society of Critical Care Medicine (SCCM), and Society of Infectious Diseases Pharmacists (SIDP). *Pharmacother. J. Hum. Pharmacol. Drug Ther.* 39 10–39. 10.1002/phar.2209 30710469 PMC7437259

[B18] Vourli S. Dafopoulou K. Vrioni G. Tsakris A. Pournaras S. (2017). Evaluation of two automated systems for colistin susceptibility testing of carbapenem-resistant *Acinetobacter baumannii* clinical isolates. *J. Antimicrob. Chemother.* 72 2528–2530. 10.1093/jac/dkx186 28605445

[B19] Zhou M. Wang Y. Liu C. Kudinha T. Liu X. Luo Y. (2018). Comparison of five commonly used automated susceptibility testing methods for accuracy in the China Antimicrobial Resistance Surveillance System (CARSS) hospitals. *Infect. Drug Resist.* 11 1347–1358. 10.2147/IDR.S166790 30214255 PMC6122890

